# Editorial: Global excellence in Toxicology: Central and South America

**DOI:** 10.3389/ftox.2024.1494491

**Published:** 2024-10-22

**Authors:** Miriam Beatriz Virgolini, Ricardo Bastos Cunha

**Affiliations:** ^1^ IFEC. CONICET, Departamento de Farmacología Otto Orsingher, Facultad de Ciencias Químicas, Universidad Nacional de Córdoba, Cordoba, Argentina; ^2^ Divisão de Química Analítica, Instituto de Química, Universidade de Brasília, Brasilia, Brazil

**Keywords:** new psychoactive substances, ethanol, corticosterone, atrazine, hexavalent chromium (Cr (VI))

In the Global Excellence in Toxicology Research Topic of Frontiers in Toxicology dedicated to studies produced in Central and South America, four papers explore various toxicants, ranging from legal and illegal substances to pesticides and metals. In addition, the toxicological effects described in these studies are examined using a range of experimental models, from primary cultures and invertebrates to vertebrates. Moreover, an article deals with the Research Topic of new psychoactive substances (NPS).

In an interdisciplinary work, involving researchers from the University of Brasilia, a pharmaceutical company, and the technical expertise of the Civil Police of the Federal District of Brazil, this article reports fatal cases involving NPS and trends in analytical techniques to characterize and quantify such substances.

In recent years, the field of toxicology has witnessed a significant evolution with the emergence of NPS. These substances, often synthesized to mimic the effects of traditional drugs, pose a growing public health challenge. NPS is often developed to circumvent existing drug laws, resulting in rapid proliferation that is difficult to control. The lack of clinical and toxicological data on these substances makes assessing their risks a significant challenge. The impact of NPS on public health is profound. Reports of intoxication, addiction, and even deaths related to the use of these substances are increasing. The scientific community is committed to better understanding the mechanisms of action of these drugs, their short- and long-term effects, and how to best treat affected individuals.

The regulation of NPS is urgent! Authorities face the challenge of identifying and banning new substances before they become widely available. Effective policies must balance the need for control with the importance of not criminalizing users, focusing on public health and harm reduction approaches.

In this scenario, scientific research plays a crucial role in responding to NPS. Laboratory, clinical, and epidemiological studies are essential to map the panorama of these substances. These substances represent a complex and multifaceted challenge. Science must work together with regulatory and public health sectors to address this emerging threat. Innovation must go hand in hand with responsibility, and the protection of public health must be the top priority.

Another article, authored by researchers from Uruguay, aims to unravel the effects of ethanol and corticosterone, both individually and in combination, on DNA damage and cell arrest, revealing complex interactions in astrocyte nuclear functionality. These results are significant in the medical context, considering ethanol as a widely available substance responsible for alcohol use disorders, and corticosterone as a hormone indicative of stress-induced dysregulation in the body. Some theories suggest that ethanol is consumed to alleviate the negative arousal caused by stress due to its anxiolytic properties (Bailey et al., 2024). Thus, in light of the results presented in the article, ethanol, and corticosterone co-existence may induce functional, though not morphological, damage to astrocytes, a critical component for central nervous system integrity.

In another turn, herbicides are widely used in agriculture to control weeds, but their extensive use has led to significant environmental contamination. These chemicals can seep into soil and water, affecting entire ecosystems. In addition to environmental impacts, some herbicides have been linked to neurotoxicity in humans and animals. Studies indicate that prolonged exposure to certain herbicides can interfere with the nervous system, causing adverse effects such as tremors, seizures, and even neurodegenerative diseases (Saravi and Dehpour, 2016). Therefore, it is crucial to monitor and regulate the use of these products to minimize their health and environmental risks. It is this neurotoxicity that researchers from the National Autonomous University of Mexico sought to study in a work included in this Research Topic. They investigated the neurotoxicity of the herbicide atrazine (ATR), one of the most widely used in the world. The researchers found that the nucleus accumbens and ventral midbrain are susceptible to repeated exposure to ATR in female rodents. However, the levels of GABA, glutamine, and glutamate remained unchanged in all brain regions evaluated, in contrast to results previously obtained with male rodents.

Finally, researchers from the Polytechnic Institute of Mexico raise the question of whether environmental modifications and short-term exposure to *Ceriodaphnia dubia* can replicate chronic toxicity to chromium. The use of invertebrates as experimental models has gained significant attention in various fields, including Toxicology. Beyond general definitions of new approach methodologies (NAMs) (see, for example, Sewell et al., 2024), invertebrates fulfill what the U.S. Environmental Protection Agency (EPA) considers a NAM as “any non-vertebrate animal technology, methodology, approach, or a combination thereof that can be used to provide information on chemical hazard and risk assessment” (EPA, 2024)^1^. However, the advantages of using simple organisms may also present pitfalls; therefore, as the authors suggest, caution should be exercised when interpreting this data in a risk assessment context. Equally important in this study is the focus on hexavalent chromium, the most toxic form of the metal, known for its diverse oxidation states, persistence in the environment, and carcinogenic effects, which altogether enhance the toxicological significance of this publication.

In summary, although rooted in ancient origins, Toxicology is a relatively new discipline that encompasses multiple areas of application. Notably, the Central and South American region is experiencing a surge in research that extends beyond the traditional clinical settings of this field, incorporating descriptive and mechanistic approaches in research laboratories. These findings often return to the clinical setting through translational toxicology, effectively bridging the gap between basic research and clinical practice.

## Footnotes

1
https://www.epa.gov/chemical-research/epa-new-approach-methods-efforts-reduce-use-vertebrate-animals-chemical-testing
